# Slope Stability Monitoring Using Novel Remote Sensing Based Fuzzy Logic

**DOI:** 10.3390/s19214636

**Published:** 2019-10-24

**Authors:** Hossein Moayedi, Dieu Tien Bui, Loke Kok Foong

**Affiliations:** 1 Department for Management of Science and Technology Development, Ton Duc Thang University, Ho Chi Minh City, Vietnam; hossein.moayedi@tdtu.edu.vn; 2 Faculty of Civil Engineering, Ton Duc Thang University, Ho Chi Minh City, Vietnam; 3 Geographic Information System Group, Department of Business and IT, University of South-Eastern Norway, N-3800 Bø i Telemark, Norway; Dieu.T.Bui@usn.no; 4 Institute of Research and Development, Duy Tan University, Da Nang 550000, Vietnam

**Keywords:** remote sensing, invasive weed optimization, slope stability monitoring

## Abstract

By the assist of remotely sensed data, this study examines the viability of slope stability monitoring using two novel conventional models. The proposed models are considered to be the combination of neuro-fuzzy (NF) system along with invasive weed optimization (IWO) and elephant herding optimization (EHO) evolutionary techniques. Considering the conditioning factors of land use, lithology, soil type, rainfall, distance to the road, distance to the river, slope degree, elevation, slope aspect, profile curvature, plan curvature, stream power index (SPI), and topographic wetness index (TWI), it is aimed to achieve a reliable approximation of landslide occurrence likelihood for unseen environmental conditions. To this end, after training the proposed EHO-NF and IWO-NF ensembles using training landslide events, their generalization power is evaluated by receiving operating characteristic curves. The results demonstrated around 75% accuracy of prediction for both models; however, the IWO-NF achieved a better understanding of landslide distribution pattern. Due to the successful performance of the implemented models, they could be promising alternatives to mathematical and analytical approaches being used for discerning the relationship between the slope failure and environmental parameters.

## 1. Introduction

The use of traditional methods for slope stability analysis has been associated with some constraints. Above all, solving problems with complicated situations is time-consuming and costly [1]. Providing design charts is known as a fundamental approach for determining the pattern of the safety factor of slopes [2,3,4,5]. Many scholars have tried to explain the relationship between a slope and its geometrical factors. One of the first studies in this subject was accomplished by Taylor [6]. He generated the slope stability design charts, which were widely used for years. Additionally, some mathematical solutions such as robust design [7,8,9,10,11] and random field theory [12,13] are still receiving considerable attention in the field of slope analysis. More recently, intelligent techniques such as the support vector machine and artificial neural network (ANN) have been broadly-used for exploring the relationship between landslide and causative factors [14,15]. The adaptive neuro-fuzzy inference system (ANFIS) is also known as a leading method for landslide susceptibility analysis [16]. Different methods of the ANFIS were tested by Polykretis, et al. [17]. Referring to the findings (Accuracy > 70%), they showed that the ANFIS is a capable tool for this purpose.

Moreover, the advent of natural-inspired metaheuristic techniques (such as artificial bee colony (ABC) and particle swarm optimization (PSO)) introduced numerous optimization opportunities to typical predictive models like ANN and ANFIS in natural hazard modelling [18,19]. In this sense, Nguyen, et al. [20] found that the ABC and PSO could act promisingly in the field of landslide modelling to enhance the performance of ANN with nearly 77% accuracy to around 80% and 86%, respectively. Similarly, Moayedi, et al. [21] used the Harris hawks optimization algorithm to increase the accuracy of the ANN in slope stability analysis. Accordingly, the performance error of the ANN was reduced by nearly 20% and 27% in terms of Root mean square error and mean absolute error, respectively. Additionally, in the case of ANFIS, many studies have conducted the optimization of this tool using hybrid evolutionary algorithms in simulating various natural phenomena like landslide, flood [22,23], groundwater spring potential [24,25] and so on. In research by Chen, et al. [26] the ANFIS achieved more than 75% accuracy for landslide susceptibility modelling when was incorporated with genetic algorithm (GA), PSO, and differential evolution techniques. Similarly, Chen, et al. [27] employed shuffled frog leaping algorithm (SFLA) and PSO to optimize the ANFIS for spatial prediction of landslide in Langao County of China. In this work, step-wise assessment ratio analysis (SWARA) was used to assess the relationship between landslide events and environmental factors. The results revealed that both ANFIS-SFLA and ANFIS-PSO models achieve the same accuracy (89%). They also found that the PSO optimizes the fuzzy system far faster than the SFLA.

The proposed methodology of the current paper comprises two major items, namely, remote sensing and metaheuristic science, for investigating the viability of slope stability monitoring. Although metaheuristic techniques have shown high robustness in natural hazard modelling, the used algorithms have been mostly selected among broadly-used ones like the PSO, imperialist competitive algorithm (ICA), and GA [28,29,30,31]. In other words, the lack of studies focusing on evaluating the optimization capability of new metaheuristic algorithms is an appreciable gap of knowledge in the mentioned field. Therefore, this research is conducted to pursue two novel objectives: (a) development of two new predictive models using invasive weed optimization and elephant herding optimization for landslide probability assessment which has not been explored in earlier studies and (b) studying a new region in a landslide-prone area of Iran.

## 2. Study Area and Data Collection

**Figure 1:**
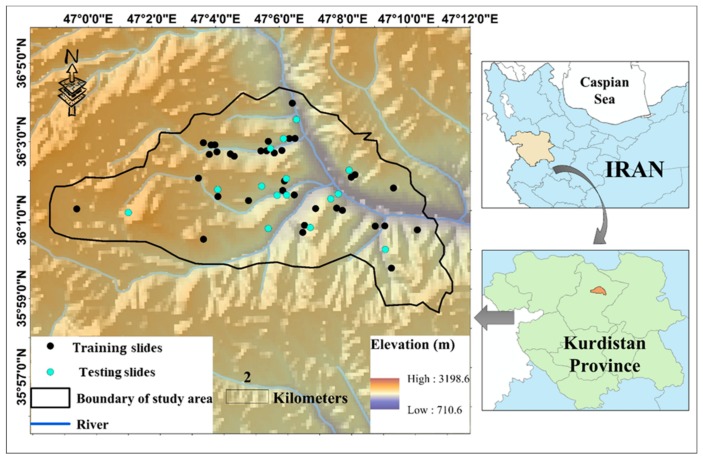
The location of the study area and the marked landslide incidents.

The location of the study area is illustrated in Figure 1. It is situated within the longitude 46°59′ to 47°11′ E and latitude 35°58′ to 36°04′ N, in the northern part of the Kurdistan Province, West of Iran. Kurdistan is a mountainous area comprising Zagros mountains. “Semi-arid with cold winters” and “temperate” are two common climates that have been mentioned in this area [32].

In this paper, utilizing remote sensing and geographic information system (GIS), the information of 13 landslide influential factors, including land use, lithology, soil type, rainfall, distance to the road, distance to the river, slope degree, elevation, slope aspect, profile curvature, plan curvature, stream power index (SPI), and topographic wetness index (TWI) were extracted. The information about the type and source of these layers is presented in Table 1. Figure 1 also shows the location of 56 historical landslides (occurred over the past 20 years). As can be seen, they have mostly occurred along with the rivers. The same number of non-landslide points are also produced within the areas devoid of landslide occurrence records. Note that the landslide susceptibility value (LSV) for the landslide and non-landslide points is considered 1 and 0, respectively. Next, out of 112 data records, 70% (i.e., 78 samples) are used for training the proposed intelligent models. The accuracy of the models is then evaluated by the remaining 30% (i.e., 34 samples).

The digital elevation model (DEM) of the Kurdistan province was created using topographic data at 1:25,000 scale. Then, the map of five conditioning factors of elevation, slope degree, profile curvature, plan curvature, and slope aspect were directly derived from the DEM. The altitude varies from around 710 to 3200 m above the sea level. The slopes are mainly gentle and their value range in [0, 25°]. Additionally, the maximum distances from the rivers and road is around 4353 and 13,346 m. The land use in the whole area is dry farming and agriculture where there are two soil categories namely, “Inceptisols” and “Rock Outcrops/Inceptisols”. The description of the geology units is also presented in Table 2. 

## 3. Methodology

**Figure 2:**
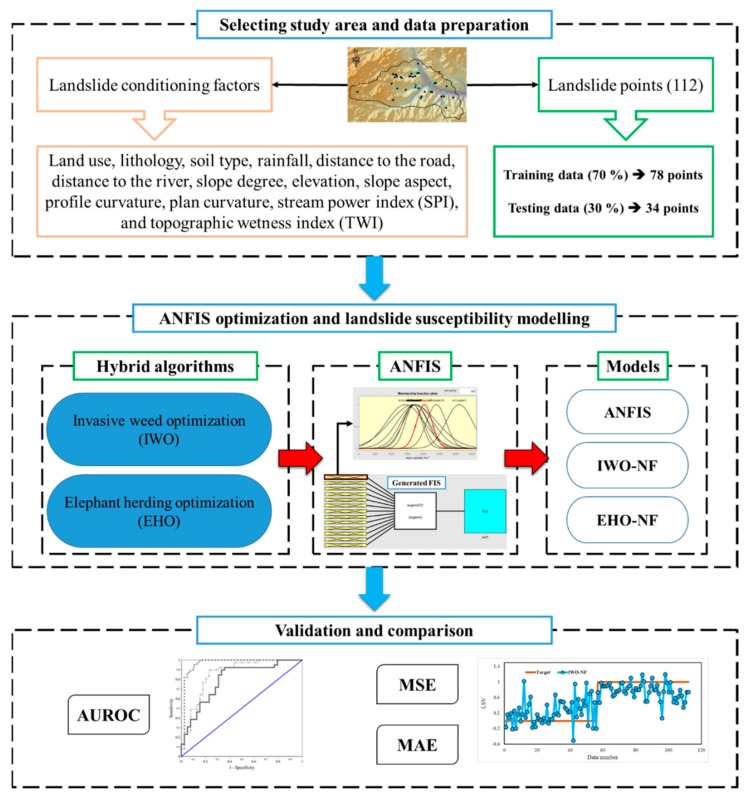
The graphical methodology of this study.

Figure 2 shows the overall steps taken for meeting the objective of this paper. After providing a proper spatial database, it was divided into the training and testing subsets. The proposed metaheuristic algorithms of the EHO and IWO were then synthesized with the ANFIS to predict the LSV. After determining the best structure of each fuzzy ensemble, the outputs were compared with actual LSVs to examine the efficiency of the applied algorithms. The results were then compared to determine the best model of the study. In this work, the accuracy of the prediction was evaluated by area under the receiving operating characteristic (AUROC) curve. The AUROC is known as a common manner for accuracy assessment of natural hazard simulation [33]. Additionally, the performance error is also measured by mean square error (MSE) and mean absolute error (MAE) criteria which are formulated as follows:

(1) $$MSE=\;\frac{1}{N}\overset{N}{\underset{i=1}{\sum }}{\left(Y_{i_{observed}\;}-Y_{i_{predicted}\;}\right)}^{2}$$

(2) $$\;MAE=\;\frac{1}{N}\overset{N}{\underset{i=1}{\sum }}\left|Y_{i_{observed}\;}-Y_{i_{predicted}\;}\right|\;$$

In the above equations, *N* represents the number of data. Moreover, *Y_i observed_* and *Y_i predicted_* denote the observed (i.e., 0 and 1) and estimated values of LSV, respectively.

The description of the used algorithms is presented in the next section.

### 3.1. Adaptive Neuro-Fuzzy Inference System

**Figure 3:**
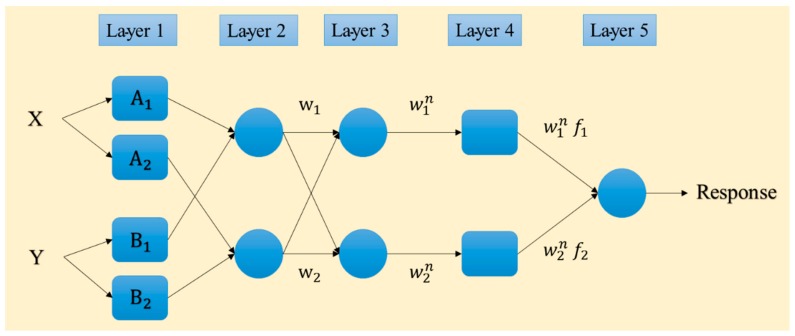
Typical structure of the ANFIS network.

Proposed by Jang [34], the adaptive neuro-fuzzy inference system (ANFIS) is based on the fuzzy theory and if-then rules. It is a robust predictive tool which benefits both ideas of neural and fuzzy computing [35]. Hence, the ANFIS has been considered to produce more consistent results compared to typical FIS. The back-propagation gradient descent and least-squares methods are combined in this tool for adjusting the membership function (MF) parameters. Figure 3 shows the structure of the ANFIS. As is seen, there are five layers. Input data (*X* and *Y*) are received by the adaptive nodes in the first layer. In the next layer, the output of each node (*W*_1_ and *W*_2_) is produced by all input signals. These products are then normalized in the third layer ($W_{1}^{n}$ and $W_{2}^{n}$). Next, using some specific parameters (also called result parameters) of the node, a function is applied to produce the output of the fourth layer ($W_{1}^{n}f_{1}$ and $W_{2}^{n}f_{2}$), and eventually, the node in the last layer gives the overall response as the sum of all input signals [22].

### 3.2. Hybrid Optimization Techniques

Two recently-developed hybrid metaheuristic algorithms, namely elephant herding optimization (EHO) and invasive weed optimization (IWO), were used to optimize the performance of the ANFIS. The EHO and IWO are population-based techniques that attempt to find the best solution for a defined problem. In this work, fine-tuning the MF parameters of the ANFIS was the main contribution of the mentioned optimization algorithms to the problem of the landslide susceptibility analysis [36]. 

In brief, the EHO was introduced by Wang, et al. [37] in 2015. This algorithm mimics the herding behavior of elephants and draws on two major stages: (a): clan updating (in which the position of the elephant is updated based on the behavior of the matriarch) and (b): separation (which describes the solitary life of the weakest elephant living in each generation). More details about the EHO algorithm can be found in previous literature [38,39]. 

Based on the colonizing behavior of weeds, the IWO was suggested by Mehrabian and Lucas [40] in 2006. In this method, the main objective is to find the optimal place for the growth and reproduction of weeds. It has been widely used for various optimization aims because of its merits such as high robustness and simple structure. Five major components including: (a) initialization, (b) reproduction, (c) spatial dispersal, (d) competitive exclusion, and (e) termination condition are considered to reproduce seeds and creating future generation. The mechanism of the IWO is well-detailed in previous works [41,42,43].

## 4. Results and Discussion

This part is composed of two major sections that report the results of the optimization process and the main findings of this paper. The main concern of this work lies in evaluating the potential of EHO and IWO evolutionary techniques in fine-tuning of ANFIS in predicting the landslide occurrence. ANFIS is a leading predictive model in the field of landslide modelling. In the literature, Oh and Pradhan [16] revealed the applicability of this tool for regional landslide susceptibility modelling at Penang Island of Malaysia. In fact, they conducted a comparative study for testing various MFs of the ANFIS. The results showed that the susceptibility maps produced by a trapezoidal, triangular, polynomial, and generalized bell MFs (accuracy ≈ 84%) had more accuracy than those produced by four Gaussian and Sigmoidal-based MFs. There are also plenty of works that have tried using metaheuristic techniques like GA and PSO for optimizing the ANFIS [27].

### 4.1. Optimizing the ANFIS Using EHO and IWO

**Figure 4:**
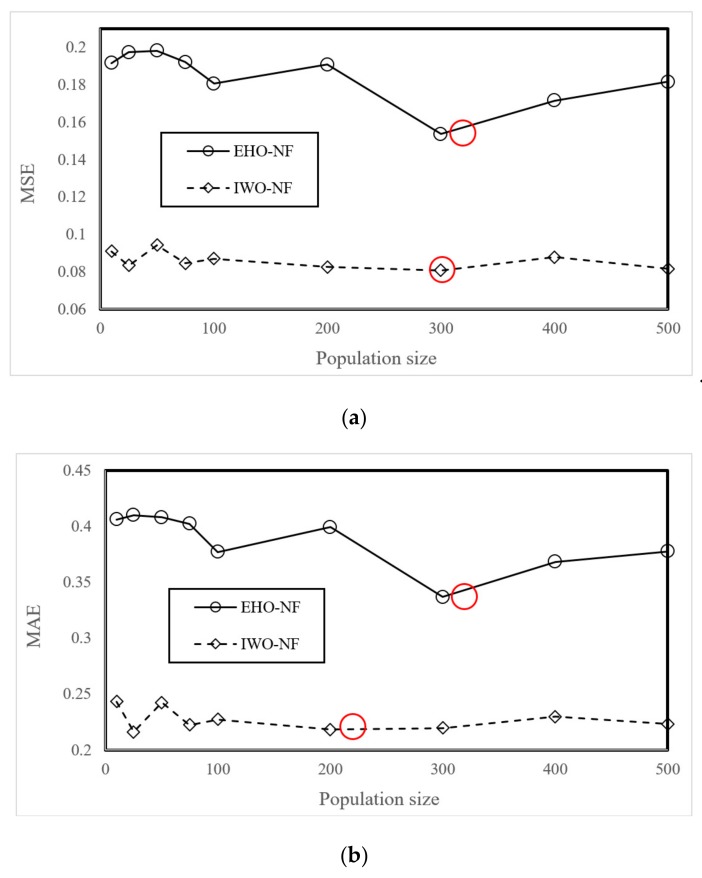
The results of the sensitivity analysis of the EHO-NF and IWO-NF based on (**a**) MSE and (**b**) MAE criteria.

The EHO and IWO algorithms were coupled with the neuro-fuzzy system using the programming language of MATLAB 2014. The outcome of this task was two fuzzy-metaheuristic ensembles of EHO-NF and IWO-NF. Next, an extensive trial and error process was conducted to determine the best-fitted structure of the used models. The models were tested within 1000 repetitions by taking into consideration nine population sizes of 10, 25, 50, 75, 100, 200, 300, 400, and 500. Remarkably, the MSE index was used as the objective function in this study. The results are presented in Figure 4a,b. These figures show the MSE and MAE obtained at the end of the 1000th iteration. As is seen, regarding the calculated value of MSE, both EHO and IWO ensembles optimized the NF by population size = 300 more efficiently than other tested values. Moreover, the calculated MAEs confirm the results for the EHO; however, a slightly lower MAE is obtained for the IWO with population size = 200. Additionally, the calculation time for the EHO-NF and IWO-NF networks obtained 4052 and 4965 s, respectively, on the operating system at 2.5 GHz and six gigabytes of RAM.

### 4.2. Model Assessment

**Figure 5:**
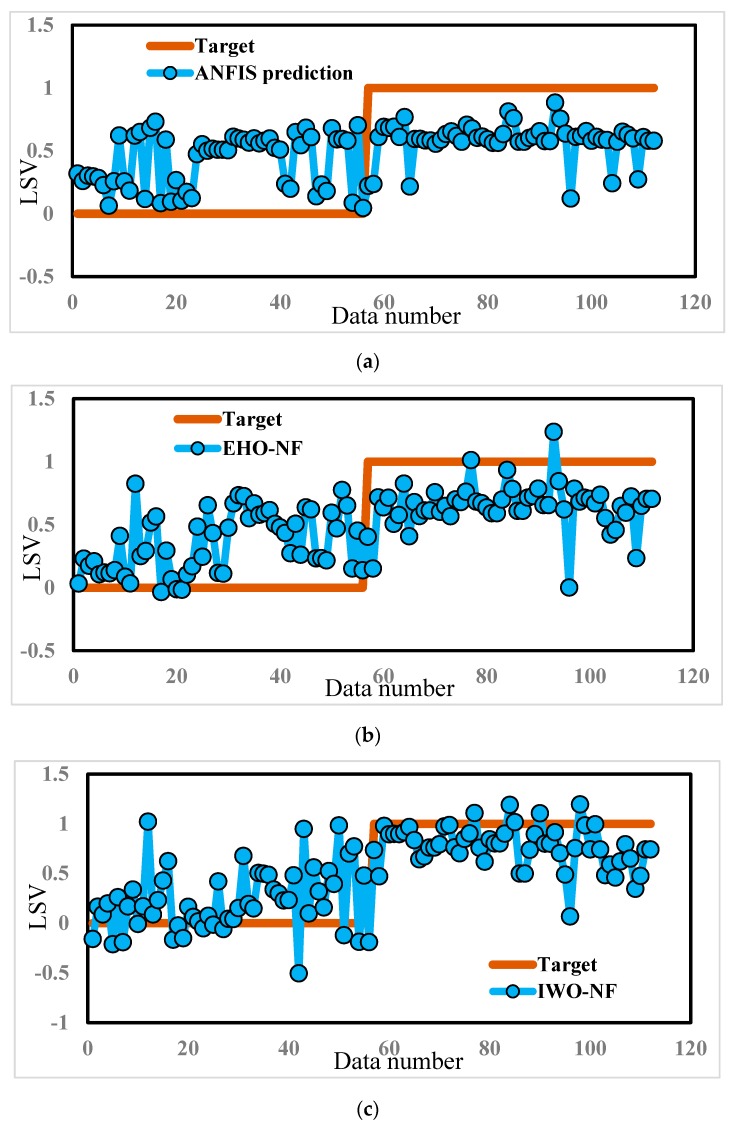
The results obtained by (**a**) typical ANFIS, (**b**) EHO-NF, and (**c**) IWO-NF for the whole dataset.

The response of each model for the training and testing samples was then extracted and compared to the expected LSVs. In other words, each one of ANFIS, EHO-NF, and IWO-NF used the training landslides to recognize the pattern between the landslide occurrence and considered conditioning factors. Then, they applied the gained knowledge to the unseen environmental conditions (i.e., the testing inputs) to estimate the LSV. Figure 5 depicts a graphical comparison between the actual and predicted LSVs, suggested by ANFIS, EHO-NF, and IWO-NF models (for the whole data).

As mentioned previously, to have a quantitative understanding of the accuracy of the models in the training and testing stages, the error of performance is calculated by means of the MSE and MAE criteria. According to these values, both proposed evolutionary algorithms showed a high capability for optimizing the ANFIS. Overall, the IWO achieved a considerably more accurate understanding of the relationship between the landslide and conditioning factors. The results of the pairwise comparison (using the method by Hanley and McNeil [44]) for the training ROC curves support this claim. Accordingly, *p*-values for the comparison between the performance of ANFIS~IWO-NF and ANFIS~EHO-NF were shown to be 0.0002 and 0.0059, respectively, which indicates a statistically significant difference between the performance of the typical and improved fuzzy networks.

**Figure 6:**
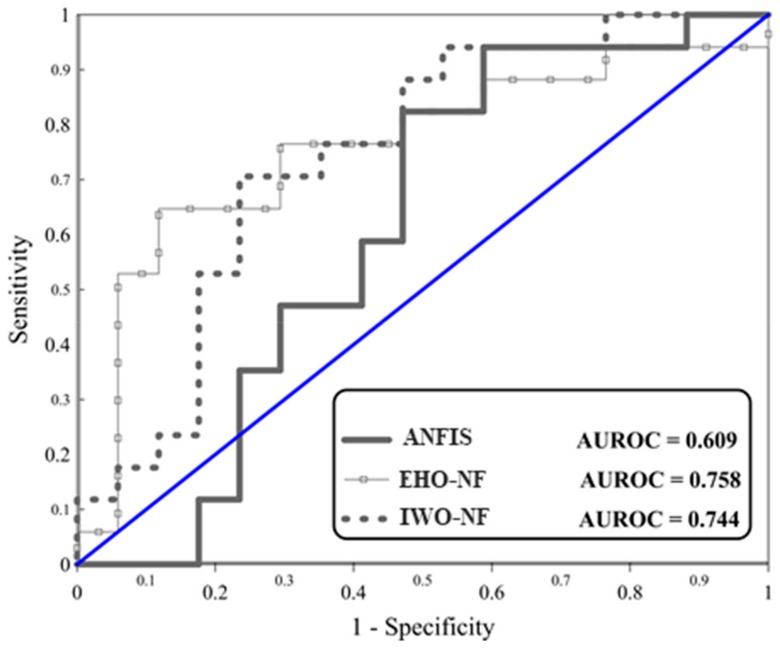
The ROC diagrams for the used models.

Regarding testing landslides, referring to the obtained MSEs (0.2380, 0.2112, and 0.2255) and MAEs (0.4457, 0.4160, and 0.4019), it can be derived that the performance error of both EHO-NF and IWO-NF is lower than the typical ANFIS. This indicates that the mentioned algorithm could enhance the generalization power of the neuro-fuzzy system. Moreover, Figure 6 depicts the ROC curves of the used models. Note that plotting this curve is a common way for evaluating the accuracy of prediction for diagnostic problems [45], and shows the specificity versus sensitivity [46,47]. According to this figure, the EHO surpasses other models as it achieved the largest accuracy of prediction (AUROC = 0.758). Additionally, the IWO-NF emerged as the second reliable model, due to its larger accuracy compared to the typical ANFIS (AUROC_ANFIS_ = 0.609 and AUROC_IWO-NF_ = 0.744). Table 3 shows the results of the statistical analysis of the results.

All in all, similar to many previous studies, it was demonstrated that synthesizing hybrid algorithms leads to improving the accuracy of the neuro-fuzzy system. Jaafari, et al. [48] showed the efficiency of GA, PSO, ICA, and SFLA for prevailing the overfitting of typical ANFIS in fire susceptibility analysis. In this work, the prediction accuracy of the ANFIS experienced around a 15% and 14% increase, which is considerably higher than what we achieved in previous research, such as [49]. Furthermore, considering 75% an acceptable level of accuracy [26], applying the IWO and EHO helped the ANFIS to achieve it from nearly 60% accuracy. 

Moreover, evaluating the results of this study reflects a discrepancy between the learning capability and generalization potential of the used hybrid ensembles. As mentioned in Section 4.2, the IWO-based model achieved the highest learning accuracy in terms of MAE and MSE. Table 4 summarizes the obtained values of the MSE, MAE, and AUROC. Based on this table, the EHO-NF performed more promisingly in the second phase. This claim can be supported by the lowest testing MSE as well as the highest AUROC. Regarding the mentioned issue, a score-based ranking system is developed in this table to determine the most efficient model. To do this, each model received three partial scores (for three accuracy indices). In this process, the more accurate the index indicates, the higher score it gains. Eventually, the summation of these reflects the overall ranking score (ORS). As the table outlines, the least ORS is obtained for the ANFIS which presented the poorest prediction. After that, the EHO (ORS = 8) emerged as the most capable algorithm of this study, followed by the IWO (ORS = 7). It should be mentioned that both ensembles used the same complexity (i.e., the population size = 300), to find the best-fitted solution.

**Figure 7:**
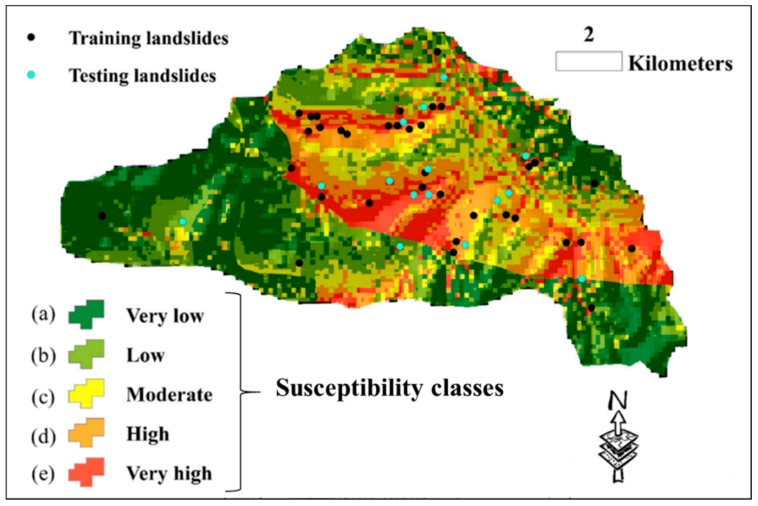
The susceptibility map of the whole area, generated by the EHO-NF model.

Lastly, the susceptibility map of the study area is generated using the elite model (i.e., the EHO-NF). To do this, the model was applied to the whole area to estimate the LSV. Then, the outputs are converted to raster in the GIS environment. Next, it was divided into five susceptibility classes (including “Very low”, “Low”, “Moderate”, “High”, and “Very high”) using the natural break classification method which is the most common method for this task [49,50,51]. The developed landslide susceptibility map is shown in Figure 7. As can be seen, the majority of landslide points have been correctly located in highly susceptible regions.

**Figure 8:**
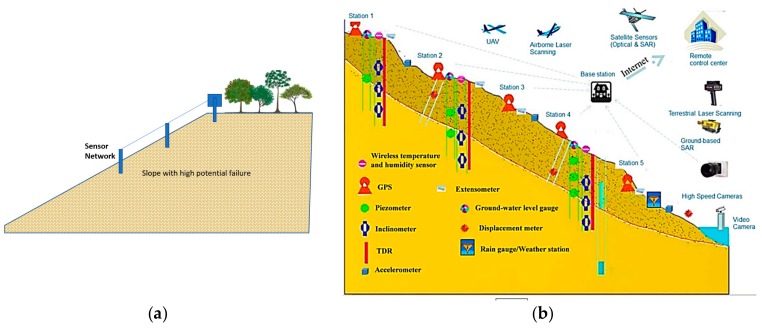
Proper location of landslide detection sensor: (**a**) column sensor installation on the slope and (**b**) an example of used sensor network for landslide early detection in Reference [56].

As Figure 7 illustrates, there are some regions in the study area that are recognized to have high landslide susceptibility. Previous studies (e.g., [52,53]) focused on the development of landslide early warning and monitoring systems; these landslide-prone areas, and especially their critical slopes, need to monitored and investigated for early prediction of landslides in order to alleviate the damages caused by this natural disaster. This process is performed using special sensors (Figure 8). In the first stage, sensors detect small motions when the sliding part is getting separated from the static one. Once the slip surface is detected, they aim to separate the subset of sensors which moved from the stabile ones. This work was carried out by conducting a distributed voting algorithm. Next, the displacement of the moved components was measured, and eventually, the position of the slip surface was estimated using the information obtained from the moved nodes [54,55].

## 5. Conclusions

Considering the importance of engineering modellings for predicting the occurrence likelihood of landslide, the main objective of this paper was to investigate and compare the applicability of two wise hybrids of ANFIS for predicting the landslide susceptibility in Western Iran. To this end, after providing the spatial dataset through remotely sensed conditioning factors, the invasive weed optimization, and elephant herding optimization algorithms are coupled with the neuro-fuzzy system in order to find the optimal parameters of its membership functions. The outcomes of the sensitivity analysis reported the value of 300 as the most suitable population size for both algorithms. Additionally, the results showed that both EHO and IWO metaheuristic algorithms could help the ANFIS to overcome computational drawbacks as the error of the ANFIS decreased by nearly 20% and 60% for the training data, and around 11% and 5% for the testing data. It was also concluded that regarding the developed ranking system in testing phase, the EHO surpassed IWO in optimizing the ANFIS in landslide probability assessment.

## Figures and Tables

**Table 1:** List and source of the spatial database.

Data Layer	Type	Source
Landslide inventory	Point	Landslide inventory database from national geoscience database of Iran (NGDIR), satellite images, and field surveys
Topographic map	Line and point	Iran’s National Cartographic Center (NCC) (Scale: 1:25,000)
Geological map	Polygon coverage	Geology Survey of Iran (GSI) (Scale: 1:100,000)
Land cover	GRID	Landsat-7 imagery
Soil map	Polygon coverage	Iranian Ministry of Agriculture-Jahad
Rainfall	GRID	Kurdistan meteorological stations

**Table 2:** The description of the lithology units.

Symbol	Description	Geological Age	Age Era
Plms	Marl, shale, sandstone, and conglomerate	Pliocene	CENOZOIC
pCmt1	Medium-grade, regional metamorphic rocks (Amphibolite Facies)	PreCambrian	PROTEROZOIC
OMql	Massive to thick-bedded reefal limestone	Oligocene-Miocene	CENOZOIC
mb	Marble	Triassic	MESOZOIC
Qft1	High-level piedmont fan and vally terrace deposits	Quaternary	CENOZOIC

**Table 3:** The results of the statistical analysis of testing ROC curves.

Methods	Area	Std. Error	p Value	Youden Index j	Asymptotic 95% Confidence Interval	
					Lower Bound	Upper Bound
**ANFIS**	0.609	0.1020	0.2867	0.3529	0.427	0.771
**EHO-NF**	0.758	0.0874	0.0032	0.5294	0.581	0.888
**IWO-NF**	0.744	0.0869	0.0050	0.4706	0.566	0.878

**Table 4:** The obtained accuracy criteria for the used models.

Model	Criterion			Score			Overall Ranking Score (ORS)	Rank
	MSE	MAE	AUROC	MSE	MAE	AUROC		
ANFIS	0.2380	0.4457	0.609	1	1	1	3	3
EHO-NF	0.2112	0.4160	0.758	3	2	3	8	1
IWO-NF	0.2255	0.4019	0.744	2	3	2	7	2
